# Author Correction: MUC1-C regulates lineage plasticity driving progression to neuroendocrine prostate cancer

**DOI:** 10.1038/s41467-020-14808-w

**Published:** 2020-02-24

**Authors:** Yota Yasumizu, Hasan Rajabi, Caining Jin, Tsuyoshi Hata, Sean Pitroda, Mark D. Long, Masayuki Hagiwara, Wei Li, Qiang Hu, Song Liu, Nami Yamashita, Atsushi Fushimi, Ling Kui, Mehmet Samur, Masaaki Yamamoto, Yan Zhang, Ning Zhang, Deli Hong, Takahiro Maeda, Takeo Kosaka, Kwok K. Wong, Mototsugu Oya, Donald Kufe

**Affiliations:** 1 Dana-Farber Cancer Institute Harvard Medical School, Boston, MA USA; 2 Department of Radiation and Cellular Oncology, University of Chicago, Chicago, IL USA; 3 Department of Biostatistics and Bioinformatics Roswell Park Comprehensive Cancer Center, Buffalo, NY USA; 4 Department of Urology, Keio University School of Medicine Shinjuku-ku, Tokyo, Japan; 5 Laura and Isaac Perlmutter Cancer Center, New York University Langone Medical Center, New York, NY USA; 6 Present Address: Department of Gastrointestinal Surgery, Graduate School of Medicine, Osaka University, Suita, Osaka, Japan

**Keywords:** Neuroendocrine cancer, Prostate cancer, Cancer stem cells, Oncogenes

Correction to: *Nature Communications* 10.1038/s41467-019-14219-6; published online 17 January 2020.

In the original version of this Article there was an error in Fig. 5. Figure 5g was inadvertently duplicated from Fig. 5f. This has now been corrected in the PDF and HTML versions of the Article.

