# Supplementary material for: MUC1-C regulates lineage plasticity driving progression to neuroendocrine prostate cancer
Source: Nat Commun. 2020 Jan 17;11:338. doi: 10.1038/s41467-019-14219-6 (PMC6969104; doi:10.1038/s41467-019-14219-6)
Supplement: Supplementary file 1 — Supplementary Info [file 41467_2019_14219_MOESM1_ESM.pdf]

MUC1-C REGULATES LINEAGE PLASTICITY DRIVING PROGRESSION TO NEUROENDOCRINE PROSTATE CANCER

Yasumizu Y., et.al.

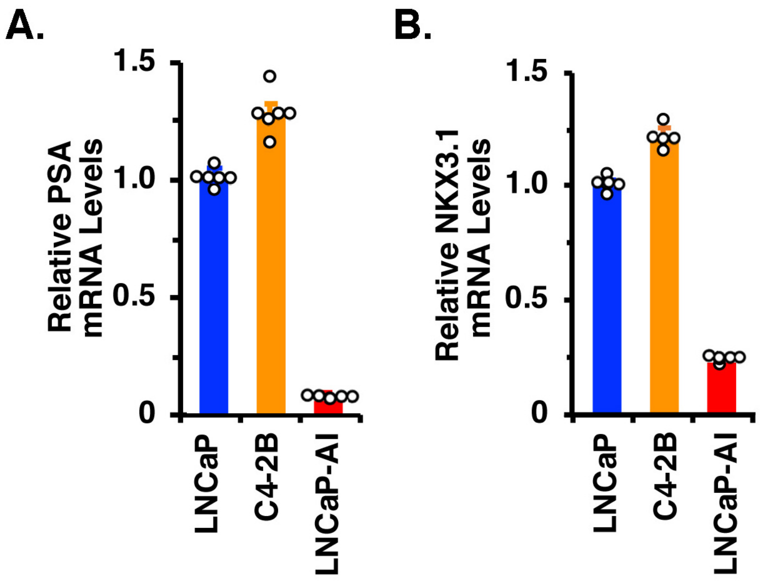

Supplementary Figure 1. Downregulation of PSA and NKX3.1 mRNA levels in LNCaP-AI cells. a and b. LNCaP, C4-2B and LNCaP-AI cells were analyzed for PSA (a) and NKX3.1 (b) mRNA levels by qRT-PCR using primers listed in Supplementary Table 1. The results (mean±SD of five determinations) are expressed as relative mRNA levels compared to that obtained for LNCaP cells (assigned a value of 1). Source data are provided as a Source Data file.

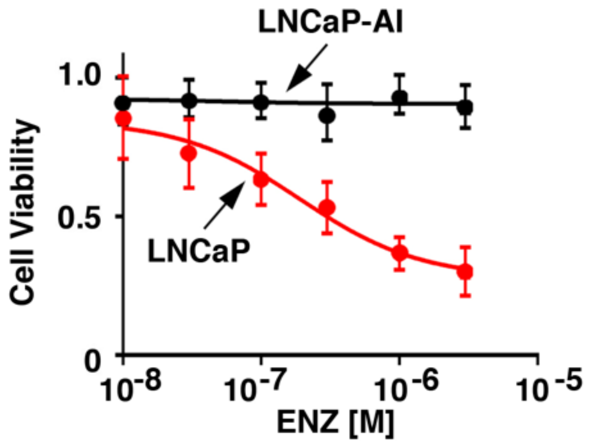

Supplementary Figure 2. LNCaP-AI cells are resistant to ENZ treatment. LNCaP and LNCaP-AI cells treated with the indicated concentrations of enzalutamide (ENZ) for 72 h were monitored for viability by trypan blue exclusion. The results (mean±SD of five determinations) are expressed as relative cell viability compared to that obtained for control untreated cells (assigned a value of 1). Source data are provided as a Source Data file.

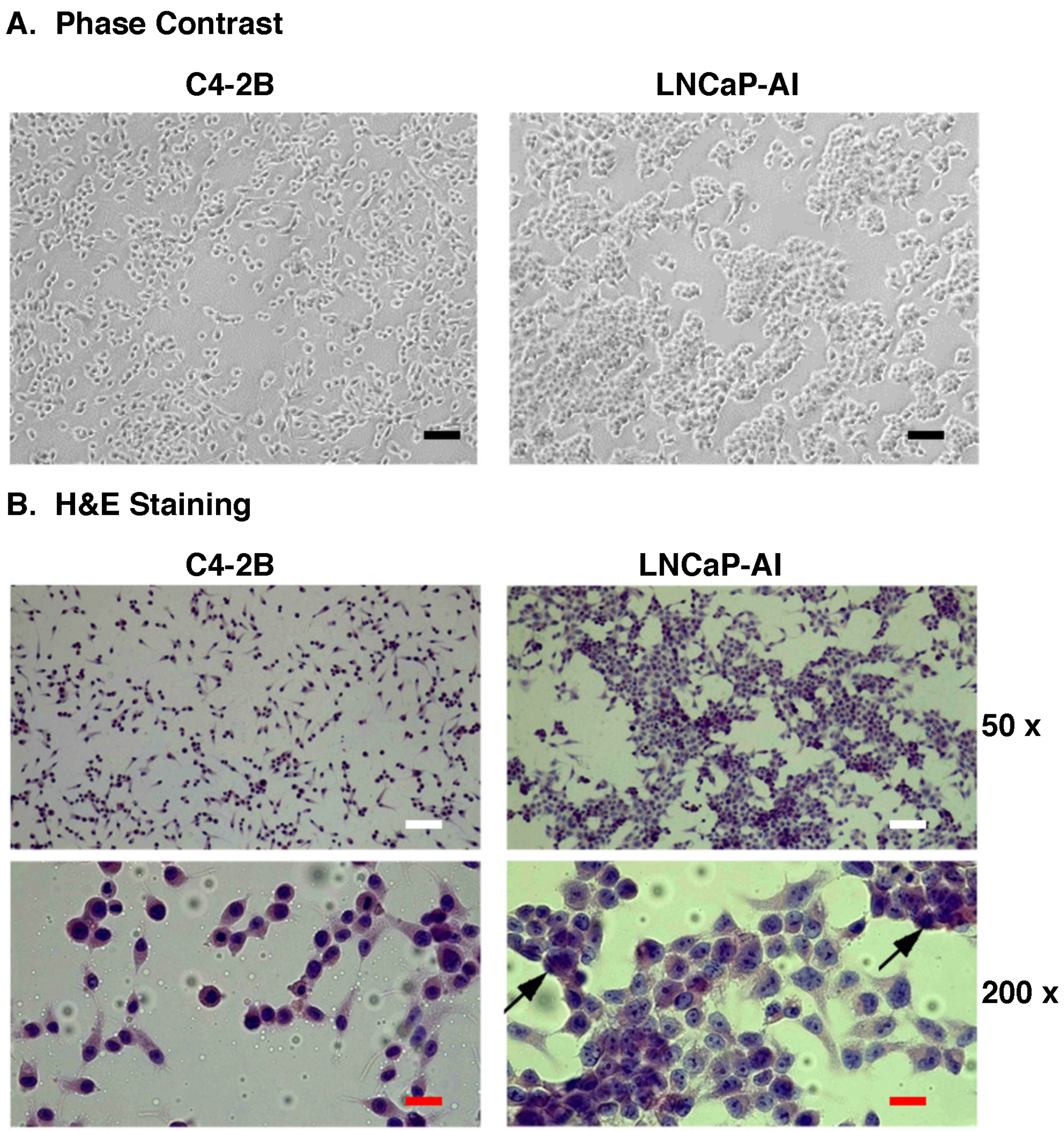

Supplementary Figure 3. Morphologic features of C4-2B and LNCaP-AI cells. a. Phase contrast visualization of C4-2B (left) and LNCaP-AI (right) cells growing as monolayers in culture. Scale bar: 100 μm. b. C4-2B (left panels) and LNCaP-AI (right panels) cells were stained with H&E and images were captured by microscopy at the indicated magnifications. Giant cells with smudgy appearing chromatin are highlighted with arrows. White scale bar: 100 μm. Red scale bar: 25 μm.

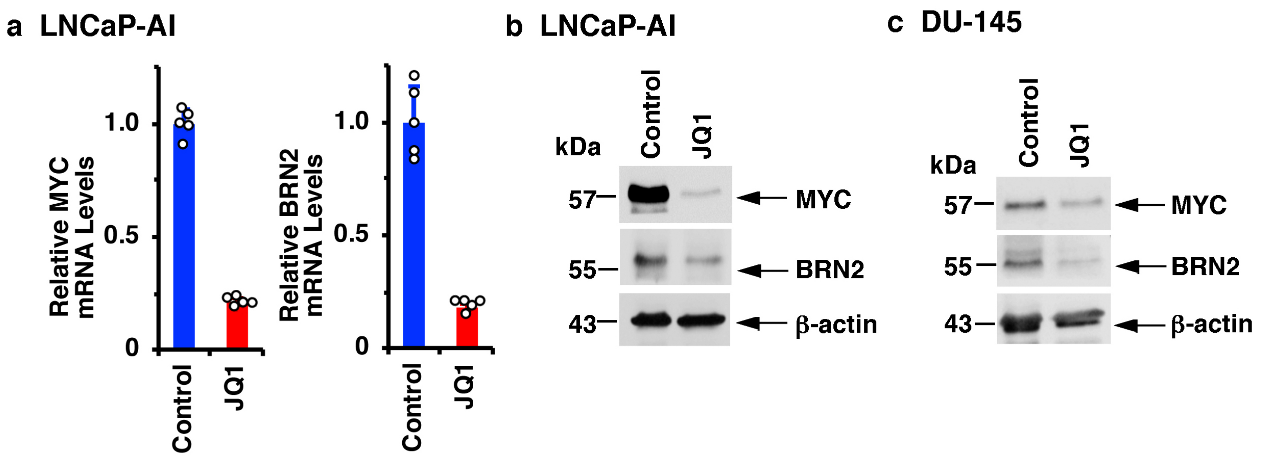

Supplementary Figure 4. Targeting MYC with JQ1 treatment suppresses BRN2 expression. a. LNCaP-AI cells treated with control vehicle (0.1% DMSO) or 5 μM JQ1 for 24 h were analyzed for MYC (left) and BRN2 (right) mRNA levels by qRT-PCR. The results (mean±SD of five determinations) are expressed as relative mRNA levels compared to that obtained for control cells (assigned a value of 1). b and c. Lysates from LNCaP-AI (b) and DU-145 (c) cells treated with control vehicle or 5 μM JQ1 for 72 h were immunoblotted with antibodies against the indicated proteins. Source data are provided as a Source Data file.

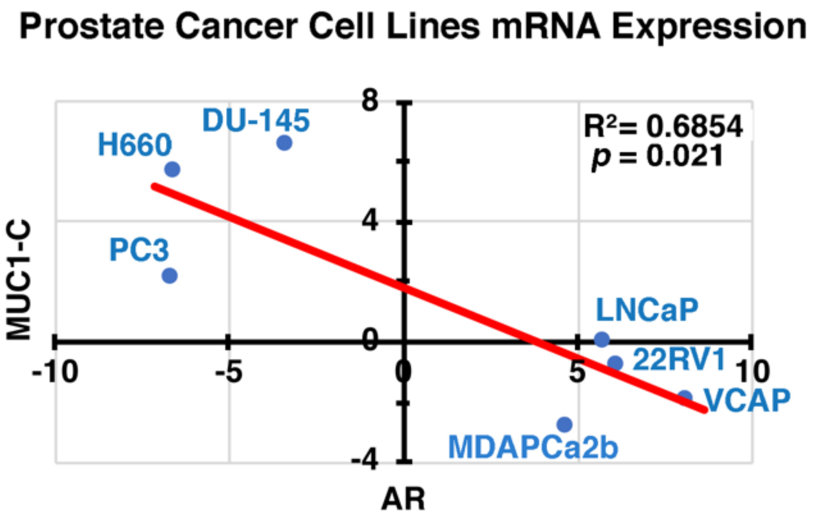

Supplementary Figure 5. Inverse correlation between MUC1 and AR expression in PC cell lines. Datasets from the "Next-generation Characterization of the Cancer Cell Line Encyclopedia" were analyzed for MUC1 and AR expression in PC cell lines.

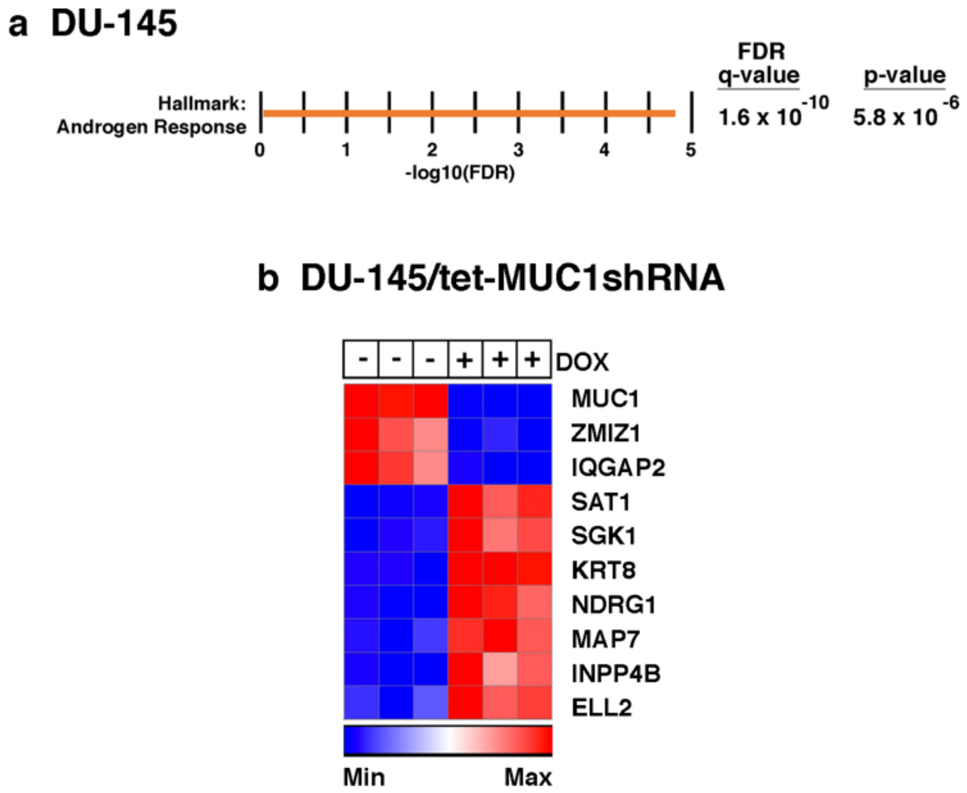

Supplementary Figure 6. MUC1-C regulates the Hallmark Androgen Response Pathway Gene Set. RNA-seq was performed in triplicate on DU-145/tet-MUC1shRNA cells treated with vehicle or 500 ng/ml DOX for 7 days. The datasets were analyzed using Hallmark for gene enrichment.

Supplementary Table 1. Primers used for qRT-PCR.

Primer	FWD	REV
GAPDH	CCATGGAGAAGGCTGGGG	CAAAGTTGTCATGGATGACC
PSA (KLK3)	CACAGCCTGTTTCATCCTGA	AGGTCCATGACCTTCACAGC
NKX3.1	GGACTGAGTGAGCCTTTTGC	CAGCCAGATTTCTCCTTTGC
MUC1	TACCGATCGTAGCCCCTATG	CTCACCAGCCCAAACAGG
EZH2	CCCTGACCTCTGTCTTACTTGTGGA	ACGTCAGATGGTGCCAGCAATA
MYC	TTCGGGTAGTGGAAAACCAG	AGTAGAAATACGGCTGCACC
AURKA	CCACCTTCGGCATCCTAATA	TCCAAGTGGTGCATATTCCA
ASCL1	CCCAAGCAAGTCAAGCGACA	AAGCCGCTGAAGTTGAGCC
SYP	TCAGTTCCGGGTGGTCAAG	AAGACCCATTGCAGCACCTT
SOX2	GAGAGAAAGAAAGGGAGAGAAG	GAGAGAGGCAAACTGGAATC
BRN2	ACACTGACCGATCTCCACGCAGTA	GAGGGTGTGGGACCCTAAATATGAC

Supplementary Table 2. Primers used for ChIP qPCR.

**ChIP-qPCR primers *BRN2* promoter**	
qF	CTGTGGCCGATAAGAGCAC
qR	CCCGATTCAGATTCTCTCAGTTC
**ChIP qPCR primer *GAPDH* promoter**	
qF	TACTAGCGGTTTTACGGGCG
qR	TCGAACAGGAGGAGCAGAGAGCGA
