# Supplementary material for: MUC1-C regulates lineage plasticity driving progression to neuroendocrine prostate cancer
Source: Nat Commun. 2020 Jan 17;11:338. doi: 10.1038/s41467-019-14219-6 (PMC6969104; doi:10.1038/s41467-019-14219-6)
Supplement: Supplementary file 3 — Reporting Summary [file 41467_2019_14219_MOESM3_ESM.pdf]

nature research

Corresponding author(s): Donald Kufe

Last updated by author(s): Dec 5, 2019

# Reporting Summary

Nature Research wishes to improve the reproducibility of the work that we publish. This form provides structure for consistency and transparency in reporting. For further information on Nature Research policies, see Authors & Referees and the Editorial Policy Checklist .

## Statistics

For all statistical analyses, confirm that the following items are present in the figure legend, table legend, main text, or Methods section.

| n/a | Confirmed | |
|---|---|---|
| ☐ | ☒ | The exact sample size ($n$) for each experimental group/condition, given as a discrete number and unit of measurement |
| ☐ | ☒ | A statement on whether measurements were taken from distinct samples or whether the same sample was measured repeatedly |
| ☐ | ☒ | The statistical test(s) used AND whether they are one- or two-sided<br>*Only common tests should be described solely by name; describe more complex techniques in the Methods section.* |
| ☒ | ☐ | A description of all covariates tested |
| ☒ | ☐ | A description of any assumptions or corrections, such as tests of normality and adjustment for multiple comparisons |
| ☐ | ☒ | A full description of the statistical parameters including central tendency (e.g. means) or other basic estimates (e.g. regression coefficient) AND variation (e.g. standard deviation) or associated estimates of uncertainty (e.g. confidence intervals) |
| ☒ | ☐ | For null hypothesis testing, the test statistic (e.g. $F$, $t$, $r$) with confidence intervals, effect sizes, degrees of freedom and $P$ value noted<br>*Give P values as exact values whenever suitable.* |
| ☒ | ☐ | For Bayesian analysis, information on the choice of priors and Markov chain Monte Carlo settings |
| ☐ | ☒ | For hierarchical and complex designs, identification of the appropriate level for tests and full reporting of outcomes |
| ☐ | ☒ | Estimates of effect sizes (e.g. Cohen's $d$, Pearson's $r$), indicating how they were calculated |

*Our web collection on statistics for biologists contains articles on many of the points above.*

## Software and code

Policy information about availability of computer code

| | |
|---|---|
| Data collection | N/A |
| Data analysis | N/A |

For manuscripts utilizing custom algorithms or software that are central to the research but not yet described in published literature, software must be made available to editors/reviewers. We strongly encourage code deposition in a community repository (e.g. GitHub). See the Nature Research guidelines for submitting code & software for further information.

## Data

Policy information about availability of data

All manuscripts must include a data availability statement. This statement should provide the following information, where applicable:

- Accession codes, unique identifiers, or web links for publicly available datasets
- A list of figures that have associated raw data
- A description of any restrictions on data availability

The RNA-seq data have been deposited in the GEO database under accession code GSE139335. (https://www.ncbi.nlm.nih.gov/geo/query/acc.cgi?acc=GSE139335).

The TCGA-PRAD, SU2C-CRPC and NEPC cohorts referenced during the study are available from the cBioPortal (http://www.cbioportal.org/ and https://www.cbioportal.org/study/summary?id=prad_su2c_2019) and from the GEO database under accession code GSE32269.

The source data underlying Figs. 1-6 and Supplementary Figs. 1, 2 and 4 are provided as a Source Data file. All other data supporting the findings of this study are available within the article and its supplementary information files and from the corresponding author upon reasonable request. A reporting summary for this article is available as a Supplementary Information file.

Requests for data and materials should be addressed to: Donald Kufe, 450 Brookline Avenue, D830, Boston, Massachusetts, 02215, 617-632-3141 Tel.,

617-632-2934 Fax, donald_kufe@dfci.harvard.edu.

# Field-specific reporting

Please select the one below that is the best fit for your research. If you are not sure, read the appropriate sections before making your selection.

- [x] Life sciences
- [ ] Behavioural & social sciences
- [ ] Ecological, evolutionary & environmental sciences

For a reference copy of the document with all sections, see nature.com/documents/nr-reporting-summary-flat.pdf

# Life sciences study design

All studies must disclose on these points even when the disclosure is negative.

| | |
|---|---|
| Sample size | Statistical analysis. Each experiment was performed at least three times from distinct samples. Data are expressed as the meanSD. The unpaired Mann-Whitney U test and Student's t-test were used to determine differences between means of groups. A p-value of <0.05 denoted by an asterisk (*) was considered statistically significant. |
| Data exclusions | Data were not excluded from analyses. |
| Replication | Attempts at replication were successful. |
| Randomization | Not performed. |
| Blinding | Not performed. |

# Reporting for specific materials, systems and methods

We require information from authors about some types of materials, experimental systems and methods used in many studies. Here, indicate whether each material, system or method listed is relevant to your study. If you are not sure if a list item applies to your research, read the appropriate section before selecting a response.

## Materials & experimental systems

| n/a | Involved in the study | |
|---|---|---|
| [ ] | [x] | Antibodies |
| [ ] | [x] | Eukaryotic cell lines |
| [x] | [ ] | Palaeontology |
| [ ] | [x] | Animals and other organisms |
| [x] | [ ] | Human research participants |
| [x] | [ ] | Clinical data |

## Methods

| n/a | Involved in the study | |
|---|---|---|
| [x] | [ ] | ChIP-seq |
| [x] | [ ] | Flow cytometry |
| [x] | [ ] | MRI-based neuroimaging |

## Antibodies

| | |
|---|---|
| Antibodies used | All information regarding antibodies is included in the "Immunoblotting" and "Chromatin Immunoprecipitation Assays" sections. |
| Validation | Validation of the antibodies was based on the manufacturer's information. |

## Eukaryotic cell lines

Policy information about cell lines

| | |
|---|---|
| Cell line source(s) | Cell line sources are included in the "Cell Culture" section. |
| Authentication | Authentication was performed by STR analysis as described in the "Cell Culture" section. |
| Mycoplasma contamination | Cells were monitored for mycoplasma contamination as described in the "Cell Culture" section. |
| Commonly misidentified lines (See ICLAC register) | None used. |

## Animals and other organisms

Policy information about studies involving animals; ARRIVE guidelines recommended for reporting animal research

| | |
|---|---|
| Laboratory animals | Information regarding mice used is included in the "Mouse tumor model studies" section. |
| Wild animals | N/A |
| Field-collected samples | N/A |
| Ethics oversight | These studies were conducted in accordance with and approval by the Dana-Farber Cancer Institute Animal Care and Use Committee (IACUC) under protocol 03-029. Included in the "Mouse tumor model studies" section. |

Note that full information on the approval of the study protocol must also be provided in the manuscript.
